# Supplementary material for: A novel artificial intelligence-based approach for identification of deoxynucleotide aptamers
Source: PLoS Comput Biol. 2021 Aug 3;17(8):e1009247. doi: 10.1371/journal.pcbi.1009247 (PMC8362955; doi:10.1371/journal.pcbi.1009247)
Supplement: Attachment — Submitted filename: Response to Reviewers.pdf [file pcbi.1009247.s001.pdf]

Response to Reviewers

Ms. Ref. No.: **PCOMPBIOL-D-20-01755**

Title: **A novel artificial intelligence-based approach for identification of deoxynucleotide aptamers**

Article Type: **Research Article**

All responses were provided according to *Reviewer Comments*. Authors want to thank reviewers for all given suggestions and questions to make this manuscript complete and clearer to readers.

<u>Reviewer #1:</u>

- abstract: you mention accuracy and auroc parameters, this is too technical and at this point these quantities are undefined and may be obscure to the general audience. Find a better way to present that information.

✓ **Response:** Definitions for both parameters were included for clarity purposes.

  - ***current version:*** Four ML algorithms (i.e., Logistic Regression, LR; Decision Tree, DT; Gaussian Naïve Bayes, GNB; Support Vector Machines, SVM) were trained using data from the NLP method along with sequence information. From four trained ML algorithms, the best performance and selected model was SVM, because it had the best discriminatory metrics (i.e., Accuracy (A)=0.995; AUROC (AU)=0.998).
  - ***revised version:*** Four ML algorithms (Logistic Regression, Decision Tree, Gaussian Naïve Bayes, Support Vector Machines) were trained using data from the NLP method along with sequence information. The best performing model was Support Vector Machines because it had the best ability to discriminate between positive and negative classes. In our model, an Accuracy (A) of 0.995, the fraction of samples that the model correctly classified, and an Area Under the Receiving Operating Curve (AUROC) of 0.998, the degree by which a model is capable of distinguishing between classes, were observed.

- line 111: provide a reference for the previous study

✓ **Response:** A reference has been included and is shown on line 131.

  - ***current version:*** The 'k' ('n') value was set to 6 because a previous study indicated that 6-mers performed better than k-mers of other lengths in target-aptamer identification.
  - ***revised version:*** The 'k' ('n') value was set to 6 because a previous study indicated that 6-mers performed better than k-mers of other lengths in target-aptamer identification [41].

- line 139: provide a definition of Accuracy, Specificity, Selectivity and AUROC metric

✓ **Response:** Definitions for Accuracy, Specificity, Selectivity and AUROC metric were included and are shown starting from line 157.

  - ***current version:*** Cross validation methods and their respective confusion matrices were used to compute Accuracy, Specificity, Selectivity and AUROC metric values, for model performance comparisons. Validations results (metric values) using the testing set were also computed and reported.
  - ***revised version:*** A set of metrics [44] was chosen for model performance comparisons including Accuracy, Specificity, Sensitivity and AUROC metric values, which are defined as follows:
    - Accuracy is the fraction of samples that the model correctly classified and is defined as (TP+TN)/(TP+FP+FN+TN), where TP is True Positive, FP is False Positive, FN is False Negative, and TN is True Negative.

- Specificity is the ratio of samples that the model correctly classified as negative classes to all the negative samples, and is defined as TN/(TN+FP).
- Sensitivity represents the ratio of samples that the model correctly classified as positives classes to all the positive samples, and is defined as TP/(TP+FN).
- Area Under the Receiver Operating Characteristics (AUROC) is a probability curve where the true positive rate is plotted against the false positive rate, the area under this curve represents degree by which a model is capable of distinguishing between classes [45].

- line 170: provide a definition of C, gamma

✓ **Response:** Definitions for C value and gamma were included and are shown starting from line 200.

- ***current version:*** A Grid search was used to tune model hyperparameters by 5-fold cross-validation. The final classifier used a C value of 10 and a gamma of 0.01.
- ***revised version:*** A Grid search with 5-fold cross-validation was used to tune model hyperparameters: C, a hyperparameter which adds a penalty for each misclassified data point and gamma, a hyperparameter which controls the level of influence of a single training point has on the model. The final classifier used a C value of 10 and a gamma of 0.01.

- line 175: provide a definition for the 'confusion matrix'

✓ **Response:** A definition for the confusion matrix was included and is shown starting from line 170.

- ***current version:*** The generated models and the confusion matrix were plotted for visual inspection.
- ***revised version:*** The confusion matrix is a tabular display of the samples by their actual and predicted class. Validations results using the testing set, as well as the confusion matrix for each model, were also computed and reported.

- line 286: table 1, too many digits in mean values taking into account the error.

✓ **Response:** Digits in mean values were fixed accordingly and are shown (in Table 1) starting from line 327.

- line 329: fig. 2: I cannot read the vertical axis of the plots even zooming in. Better reduce the number of shown cases to most representative ones.

✓ **Response:** Fig 2 was changed showing the most relevant variables only. The new caption of Fig 2 is shown starting on line 577.

- line 351: fig. 6: what is meaning of grey shaded area ?

✓ **Response:** A better description of both shaded areas was included in the caption of Fig 6 as shown starting on line 589.

- **current version:** Fig 6. Scatter plots of each ML model. DNA aptamer sequences are shown as orange dots, DNA sequences are shown as dark blue dots. The insert shows the confusion matrix of each model. (A) Logistic Regression, (B) Decision Tree Classifier, (C) Gaussian Naïve Bayes and (D) Support Vector Machines.
- **revised version:** Fig 6. Scatter plots of each ML model. DNA aptamer sequences are shown as orange dots DNA sequences are shown as dark blue dots. The insert shows the confusion matrix of each model. (A) Logistic Regression, (B) Decision Tree Classifier, (C) Gaussian Naïve Bayes and (D) Support Vector Machines. The light gray area is the boundary for predicted DNA sequences and the dark gray area is the boundary for predicted DNA aptamer sequences.

Reviewer #2:

1. I think the introduction should be improved. While the section provides excellent background on aptamer biology and SELEX, it does not mention any previous works using computational approaches for aptamer prediction, including existing 2D/3D structural approaches. A brief survey of the field on the computational side may be necessary.

- ✓ **Response:** A new paragraph related to previous works using computational approaches for aptamer prediction has been included and can be found in the introduction starting from line 84.
  - **revised version:…** Interest in the use of statistical methods in aptamer prediction approaches has grown lately. Computational techniques are simple, time-saving, cost-effective, and do not require specialized resources [34]. Aptamer's computational prediction methods have been carried out in two major categories: prediction based on interaction and prediction based on structure. Computational prediction models based on interaction, take into account the physicochemical, energetic and conformational properties of the aptamers. These models, while may not be very accurate, may shed some light on in-depth understanding of the mechanisms of interactions between aptamers and their targets, but cannot be applied to the SELEX pipeline to reduce the number of steps [35]. Computational prediction models based on structure folding tend to be more accurate, but their use is hampered by the dependency on the availability of homologous sequences [36]….

2. In the training data, the DNA sequences (negative samples) were substantially shorter than the aptamer sequence. In the pairwise scatter plot in figure 2, even just the length along is sufficient to separate aptamer from non-aptamer sequence. Clear decision boundaries can also be drawn for other features. I'm wondering if this alone gives good model performance. I think it would be more convincing if the authors could train/test on non-aptamer DNA sequences with similar sequence/length to the aptamer sequence or perhaps using randomized sequences to make sure the model is non-trivial and could be applied for harder/real-world problem.

- ✓ **Response:** Although the distribution for the length variable is quite different between both DNA sequences, during the dimensionality reduction step, the recursive elimination algorithm is capable to remove this variable. Therefore, it was deleted as an input for these models. Our best interest is to find key differences between DNA aptamers and genomic DNA. Another possible explanation of why these DNA sequences were chosen is because that although genomic, they can bind targets in a non-aptameric fashion.

3. Along the same line, I think it would be more impactful if the authors could also develop a machine learning model to predict positive aptamer sequences given a binding target, not simply just reducing the candidate pool.

- ✓ **Response:** This is a great suggestion. In fact, we are working on a new ML model to predict possible aptamer binding pairs, which will be considered for publication in the near future.

4. In figure 4, the profile of DNA vs aptamer sequence shows a very different distribution. However, these features were pre-selected based on the frequency not by the machine learning models. Therefore, it is unclear to me how these features contribute to the model performance and their relevance to the developed models. I would recommend perhaps training a regularized linear model such as elastic net to see if some of these k-mers features could be picked up by the model.

- ✓ **Response:** As part of a machine learning model, k-mer features were initially reduced by frequency on a very first step, and later, were reduced even further using recursive feature elimination. This protocol has been used by other authors, where NLP were also used to study DNA sequences.

5. How were the top 33 k-mer ranked/identified? The principal component analysis does not predict feature importance. Also, how were the top predictive features in table 4 determined? It is a bit confusing because the input to the model seems to be PCA components according to Figure 1.

- ✓ **Response:** Thank you for this note because it was indeed an error. PCA was not used to identify the top features. We used instead the Recursive Feature Elimination with Logistic regression as the estimator, as described starting from line 143.
  - **revised version:** Before modeling, dimensionality reduction was performed on the remaining features using recursive feature elimination using logistic regression as the estimator [42].

6. How was the PCA analysis look like for the data? Although PCA was used for feature reduction, tSNE was shown instead.

- ✓ **Response:** The PCA analysis was not done on our data. We used tSNE instead.

7. In table 3, the authors compared their machine learning model performance to several others existing machine learning approaches on aptamer predictions. However, difference training/testing dataset were used. While informative, I think it will be more relevant if the same dataset could be used for comparison.

- ✓ **Response:** This is another great suggestion. We are now using the generated data in another ML model, but the proposed aims are completely different from those presented in this manuscript. Our models aim to predict aptamer-target pairs from potential aptamer sequences out of crude DNA sequences. We could not be able to achieve this goal using the same data.

8. What is the scatterplot in figure 6? The x axis shows aptamer, y axis shows DNA but each dot could be either aptamer or DNA.

- ✓ **Response:** In Fig 6, both x and y axes are independent of each other and their scale uses arbitrary numbers. The light gray area correlates with the boundary that the model has predicted to be DNA sequences and the dark gray area correlates with the boundary that the model has predicted to be DNA aptamer sequences. Both descriptions have been included in Fig 6 for clarity purposes, as shown starting on line 589.
  - **current version:** Fig 6. Scatter plots of each ML model. DNA aptamer sequences are shown as orange dots, DNA sequences are shown as dark blue dots. The insert shows the confusion matrix of each model. (A) Logistic Regression, (B) Decision Tree Classifier, (C) Gaussian Naïve Bayes and (D) Support Vector Machines.
  - **revised version:** Fig 6. Scatter plots of each ML model. DNA aptamer sequences are shown as orange dots DNA sequences are shown as dark blue dots. The insert shows the confusion matrix of each model. (A) Logistic Regression, (B) Decision Tree Classifier, (C) Gaussian Naïve Bayes and (D) Support Vector Machines. The light gray area is the boundary for predicted DNA sequences and the dark gray area is the boundary for predicted DNA aptamer sequences.

9. The conclusion is quite brief. The authors could maybe put their approach into broader content to see how their approach could be developed for aptamer design. Perhaps also compare/contrast to existing approaches, such as those outlined in Table 3 to showcase the unique value/limitation of the approach.

- ✓ **Response:** The conclusion was expanded accordingly. The new conclusion is shown starting from line 356.

10. There is no implementation/code to the model. Could the authors provide perhaps with the github page along with training/testing dataset?

- ✓ **Response:** An account was created in GitHub and referenced in the manuscript as [39].

- **current version:** The codes for the python scripts and the raw data are available at Mendeley Data [1].
- **revised version:** The codes for the python scripts in GitHub [39] and the raw data are available at Mendeley Data [1].

11. Figure quality should be substantially improved and revised for better presentation. The font in figure 2 is quite small and is barely readable.

✓ **Response:** Fig 2 was modified to show the most relevant variables. The new caption of Fig 2 is shown starting from line 577.

12. Line 202, "Error! Reference source not found".

✓ **Response:** Thank you for this note because it was indeed an error. The correction was made and is shown on line 235.

- **current version:** Other features/variables chosen for the DNA and aptamer sequences used in this study are shown in **Error! Reference source not found**.
- **revised version:** Other features/variables chosen for the DNA and aptamer sequences used in this study are shown in Table 1.

13. Line 253, AUROC are 96.3% and 0.98.

✓ **Response:** Although it is not clear what the reviewer's #2 concern is, it could be useful to point out that the complete sentence reads as follows: "For LR obtained model, the accuracy and AUROC are 96.3% and 0.988, respectively."

Reviewer #3:

Comments and local corrections:

(1) Data sources. The work used 4,885 protein-binding DNA sequences and 238 aptamer sequences as datasets. These two types of data differ significantly in sequence length. Is it suitable to use them as a dataset, and what the reason for this choice?

✓ **Response:** Although the distribution for the length variable is quite different between both DNA sequences, during the dimensionality reduction step, the recursive elimination algorithm is capable to remove this variable. Therefore, it was deleted as an input for these models. Our best interest is to find key differences between DNA aptamers and genomic DNA. Another possible explanation of why these DNA sequences were chosen is because that although genomic, they can bind targets in a non-aptameric fashion.

(2) In Table 1, there are two p-values; what is the difference between them? Why do they have such a big gap?

✓ **Response:** The first p-value is between the genomic DNA and aptamer classes, while the second p-value is between the training and testing set. The difference specifies that genomic DNA and aptamer classes are different, meanwhile the training and testing set are not different.

(3) In the given URL, we could not find the codes for the python scripts.

✓ **Response:** The code was deposited on GitHub and referenced in the manuscript as [39]; https://github.com/eipm-uprm/Aptamer-ML

(4) Page 5, in the caption of Figure 1, we think the following data should be examined: the number of vectors 4080? and the number of the training set is 7,816? And the test set is 1,954?

- ✓ **Response:** Thank you for this note because it was indeed an error. The caption of Fig 1 was revised and corrected accordingly. The new caption of Fig 1 is shown starting from line 568.
    - **current version: Fig 1**. **Overview of the AI approach used to obtain a model for the classification of a sequence as an aptamer.** It included the extraction of nucleotide sequences from the Nucleic Acid Database (NDB) and Aptagen. The sequences were converted into 6-mer vectors using the NLP modules. Out of the 4,080 vectors created, only the top 2.5% were selected for modeling, in the reduction of dimensionality module. Then the data was split into a training set (80% of the data, n = 7,816) and test set (20% of the data, n = 1,954). Because of data imbalance in the training set, the underrepresented samples were weighted highly. ML algorithms were trained to develop the models using the selected features. The developed models were tested using cross-validation and validated using the test sets.
    - **revised version: Fig 1. Overview of the AI approach used to obtain a model for the classification of a sequence as an aptamer.** It included the extraction of nucleotide sequences from the Nucleic Acid Database (NDB) and Aptagen. The sequences were converted into 6-mer vectors using the NLP modules. Out of the 5,123 vectors created, only the top 2.5% were selected for modeling, in the reduction of dimensionality module. Then the data was split into a training set (80% of the data, n = 4,099) and test set (20% of the data, n = 1,024). Because of data imbalance in the training set, the underrepresented samples were weighted highly. ML algorithms were trained to develop the models using the selected features. The developed models were tested using cross-validation and validated using the test sets.

(5) Page 6, line 111-112, the references of the previous study in the sentence "…as set to 6 because a previous study indicated that 6-mers performed better than k-mers of other …" did not cite.

- ✓ **Response:** A reference was included accordingly and is found on line 131.
    - **current version:** The 'k' ('n') value was set to 6 because a previous study indicated that 6-mers performed better than k-mers of other lengths in target-aptamer identification.
    - **revised version:** The 'k' ('n') value was set to 6 because a previous study indicated that 6-mers performed better than k-mers of other lengths in target-aptamer identification [41].

(6) Page 10, line 202, Error! Reference source not found.

- ✓ **Response:** Thank you for this note because it was indeed an error. The correction was made and is shown on line 235.
    - **current version:** Other features/variables chosen for the DNA and aptamer sequences used in this study are shown in **Error! Reference source not found**.
    - **revised version:** Other features/variables chosen for the DNA and aptamer sequences used in this study are shown in Table 1.

(7) The expression of some terms is inconsistent, and this may confuse the readers. For instance, DT or DTC for Decision Tree.

- ✓ **Response:** Thank you for this note. This term and its corresponding abbreviation (DT) have been checked and corrected for consistency.
