# Supplementary material for: A novel artificial intelligence-based approach for identification of deoxynucleotide aptamers
Source: PLoS Comput Biol. 2021 Aug 3;17(8):e1009247. doi: 10.1371/journal.pcbi.1009247 (PMC8362955; doi:10.1371/journal.pcbi.1009247)
Supplement: Attachment — Submitted filename: Response to Reviewers.pdf [file pcbi.1009247.s002.pdf]

Response to Reviewers

Ms. Ref. No.: **(PCOMPBIOL-D-20-01755R1) - [EMID:bf3de20fcc7396cc]**

Title: **A novel artificial intelligence-based approach for identification of deoxynucleotide aptamers**

Article Type: **Research Article**

All responses were provided according to *Reviewer Comments*. Authors want to thank **reviewer #2** for all given suggestions and questions to make this manuscript complete and clearer to readers.

Reviewer #2:

1. It is unclear to me how the recursive feature elimination step would automatically remove length as a feature as sequence length is a very prominent feature in differentiating DNA vs aptamer classes (figure 2). Similarly, It is confusing that the length is still listed as a feature in table 1. I would recommend removing length feature from consideration before the feature elimination step and re-estimate the performance.

- ✓ **Response:** The sequence length, as one of the previous features used for this research, was removed from the input. This change had no effect on the performance of the algorithms. Table 1, Fig 2, Fig 3 and Fig 4 have been updated to show the new input variables. In "Baseline characteristics" minor changes to both paragraphs, as shown in lanes 235-237 and 247-249 in the highlighted version of the manuscript, were adapted accordingly.

2. Similarly, was Figure 2 generated with length as input?

- ✓ **Response:** The sequence length, as one of the previous features used for this research, was removed and Fig 2 was updated.

3. What is the number on the axis for figure 6?

- ✓ **Response:** Both x and y axes are arbitrary numbers, independent of each other. The labels used for both axis were removed and Fig 6 was updated.

4. The font is too small for figure 5, 6 and 8.

- ✓ **Response:** The font for all figures were corrected accordingly.

5. In figure 3, the axis labels should be tsne1 and tsne2.

- ✓ **Response:** Thank you for this note because it was indeed an error. The axis labels were corrected accordingly.

6. Resolution should be improved for all figures.

- ✓ **Response:** All figure files were uploaded to the Preflight Analysis and Conversion Engine (PACE) digital diagnostic tool, https://pacev2.apexcovantage.com/, to ensure that meets PLOS requirements.

7. Other minor grammar errors were also corrected, as follows:

Lane 357	in vitro → *in vitro*
Lane 579	represents → depicts
Lane 580	represents → depicts
Lane 586	the word "and" was added to the sentence
